# Sorption of Cu(II) Ions on Chitosan-Zeolite X Composites: Impact of Gelling and Drying Conditions

**DOI:** 10.3390/molecules21010109

**Published:** 2016-01-19

**Authors:** Amal Djelad, Amine Morsli, Mike Robitzer, Abdelkader Bengueddach, Francesco di Renzo, Françoise Quignard

**Affiliations:** 1 Institut Charles Gerhardt, UMR 5253 CNRS/UM2/ENSCM/UM1, Matériaux Avancés pour la Catalyse et la Santé, ENSCM, 8 rue Ecole Normale, 34296 Montpellier Cedex 5, France; djelad_am@yahoo.fr (A.D.); mike.robitzer@enscm.fr (M.R.); direnzo@enscm.fr (F.R.); 2 Laboratoire de Chimie Des Matériaux, Département de Chimie, Faculté des Sciences Exactes et Appliquées, Université Oran 1-Ahmed Benbella, B.P. 1524 Elmenaouar, Oran 31000, Algeria; abengueddach@gmail.com; 3 Faculté de Chimie, Université des Sciences et de la Technologie d’Oran-Mohamed Boudiaf, USTO, B.P. 1505 Elmenaouar, Oran 31000, Algeria; amine_morsli@yahoo.fr

**Keywords:** biomass, chitosan, zeolite X, composites, aerogel, metal sorption

## Abstract

Chitosan-zeolite Na-X composite beads with open porosity and different zeolite contents were prepared by an encapsulation method. Preparation conditions had to be optimised in order to stabilize the zeolite network during the polysaccharide gelling process. Composites and pure reference components were characterized using X-ray diffraction (XRD); scanning electron microscopy (SEM); N_2_ adsorption–desorption; and thermogravimetric analysis (TG). Cu(II) sorption was investigated at pH 6. The choice of drying method used for the storage of the adsorbent severely affects the textural properties of the composite and the copper sorption effectiveness. The copper sorption capacity of chitosan hydrogel is about 190 mg·g^−1^. More than 70% of this capacity is retained when the polysaccharide is stored as an aerogel after supercrititcal CO_2_ drying, but nearly 90% of the capacity is lost after evaporative drying to a xerogel. Textural data and Cu(II) sorption data indicate that the properties of the zeolite-polysaccharide composites are not just the sum of the properties of the individual components. Whereas a chitosan coating impairs the accessibility of the microporosity of the zeolite; the presence of the zeolite improves the stability of the dispersion of chitosan upon supercritical drying and increases the affinity of the composites for Cu(II) cations. Chitosan-zeolite aerogels present Cu(II) sorption properties.

## 1. Introduction

Chitosan, a linear copolymer of linked β-(1,4)-glucosamine molecules easily obtained from renewable resources, is an effective sorbent for metal species [1,2,3,4]. More specifically, chitosan has been shown to be an effective sorbent for Cu(II) cations in aqueous solution [5,6]. Chitosan is mainly obtained by deacetylation of chitin (poly-β-(1,4)-acetylglucosamine) obtained from wastes of the seafood industry (crab and shrimp shells and squid pens) and presents all the advantages of a low-cost renewable raw material [7,8,9]. Quite high-grade chitosan can be found on the market at less than 8 $/kg [10] and it therefore competes favourably with the cost of synthetic ion-exchange resins [11]. The chitosan market is relatively large, as its ease of physical and chemical modification [12,13] renders it useful in fields as varied as medical devices [14,15,16], wastewater treatment [17,18] and catalysis [19,20,21,22,23].

Biodegradability is an additional property of chitosan. This property may be interesting for some applications such as the preparation of copper chelates for the treatment of plant diseases [24]. In this case, the progressive degradation of chitosan allows a controlled release of copper. However, biodegradability may also be a serious drawback for long-time applications in sorption processes. Biodegradability in moist environment is at the basis of the requirement for storage of the adsorbent under dry conditions before its rehydration for use in aqueous solutions. The drying methods have to be optimised if a high surface area adsorbent is the target of the preparation, as evaporative drying induces a largely irreversible loss of the highly dispersed state of chitosan hydrogels [25,26,27]. Drying of chitosan has dramatic effects on Cu(II) sorption, as freshly-prepared chitosan hydrogels present a Cu(II) capacity as high as 150 mg·g^−1^ [28], while evaporatively dried chitosan xerogels have a Cu(II) capacity nearer to 20 mg·g^−1^ [5,29].

The gelling properties of chitosan allow it to be shaped in geometries and sizes more adapted to each specific use [30,31,32,33]. Its suitability as a matrix for the formation of zeolite beads has been recognized [34] and the investigation of chitosan-inorganic composites has given rise to a prolific field of research aimed at applications in the fields of medical devices [35], pollution control [36], catalysis [37,38,39], photoluminescence [40,41] and membrane separation [42]. 

Among all kinds of inorganic fillers, zeolites are especially appealing due to their thermal and chemical stability and great potential for the separation of ions by cation exchange. Zeolites are crystalline microporous aluminosilicates represented by the empirical formula M^n+^_2/n_O_2_·Al_2_O_3_·xSiO_2_·yH_2_O [43], where M are usually alkaline or alkaline earth cations. The isomorphic substitution of Si^4+^ by Al^3+^ induces the formation of lattice anions which are compensated by exchangeable cations [44]. The cation exchange capacity of the zeolites therefore depends on the framework Si/Al ratio and decreases with an increase of the Si/Al ratio. Although specialty zeolites for catalysis and gas separation are high-value added products, low Si/Al zeolites for cation exchange are commodities available on the market at prices of less than 2 $/kg [45].

Chitosan-zeolite composites have shown good adsorption properties for different pollutants such as dyes, phosphates, nitrates, ammonium, and humic acids [46,47,48,49,50] as well as for the removal of heavy metal cations [51,52,53]. Recently, chitosan-zeolite composites have been developed in membrane form for direct methanol fuel cells [54,55] and several pervaporation separations, like toluene/alcohol [56], dimethyl carbonate/methanol [57], alcohol/water [58,59,60] and water/tetrahydro-furan [61].

The preparations of chitosan-zeolite composites reported in the literature essentially follow two routes: (i) *in situ* formation of inorganic particles within a porous polymer matrix through sol–gel method [34,50]; (ii) encapsulation by suspension of inorganic fillers in a polysaccharide solution before gelling [5,62,63]. This last method was followed in the present work.

Despite the attention given to the properties of composites, the way in which the preparation of mixed systems affects the properties of each component has been less deeply delved. The stability of the zeolites in the conditions of encapsulation is a main issue not always taken in due account in the literature. In this work, a method suitable for the preparation of chitosan composites including a stable aluminium-rich zeolite Na-X is proposed.

Attention has also been given to the obtention of mesoporous composites. The porosity requirements of systems for metal removal from large volumes of solution differ from the requirement of pervaporation membranes. The decrease of head losses and the increase of uptake of metals in packed bed systems require preserving the porosity of the chitosan gels. With this objective, several procedures of drying have been compared, *viz.*, CO_2_ supercritical drying and direct solvent evaporation.

## 2. Results and Discussion

### 2.1. Synthesis and Characterization of Chitosan and Chitosan-Zeolite Composites

The simplest way to prepare a chitosan entangled hydrogel is the solubilization of chitosan in an acidic aqueous medium followed by gelling in an alkaline solution or by evaporative gelling for some membrane preparation. Both acid and alkaline treatments can affect the stability of the zeolite phase. The Si–O–Al bonds in the zeolite framework can be easily hydrolysed at low pH, whereas high-silica zeolites are highly soluble in concentrated alkali solutions. As a consequence, the choice of elaboration conditions of zeolite-chitosan composites are critical to the phase behaviour of the inorganic component, as witnessed by the X-ray diffractograms of composites reported in the literature. 

Typically, encapsulation methods implying a long permanence of the zeolite in highly acidic solutions (acidic acid/glucosamine molar ratios higher than 4) lead to the disappearance of the X-ray diffraction bands of aluminium-rich zeolites like zeolite A [54,64] or zeolite X [65], whereas more silica-rich ZSM-5 or zeolite beta better withstood the treatment [54,57,66]. When zeolites were incorporated under slightly less severe conditions (acetic acid/glucosamine mole ratios between 2 and 3), mordenite with Si/Al 6.5 withstood the treatment [67]. When zeolite Y with Si/Al 2.5 was incorporated under the same conditions [68], partial preservation of the zeolite did not prevent the appearance of the typical broad hump of amorphous aluminosilicates around 30°2ϴ [69,70] in the X-ray diffractogram and a significant high-angle shift of the zeolite DRX peaks was observed, corresponding to a dealumination of the zeolite [71,72,73].

**Figure 1:**
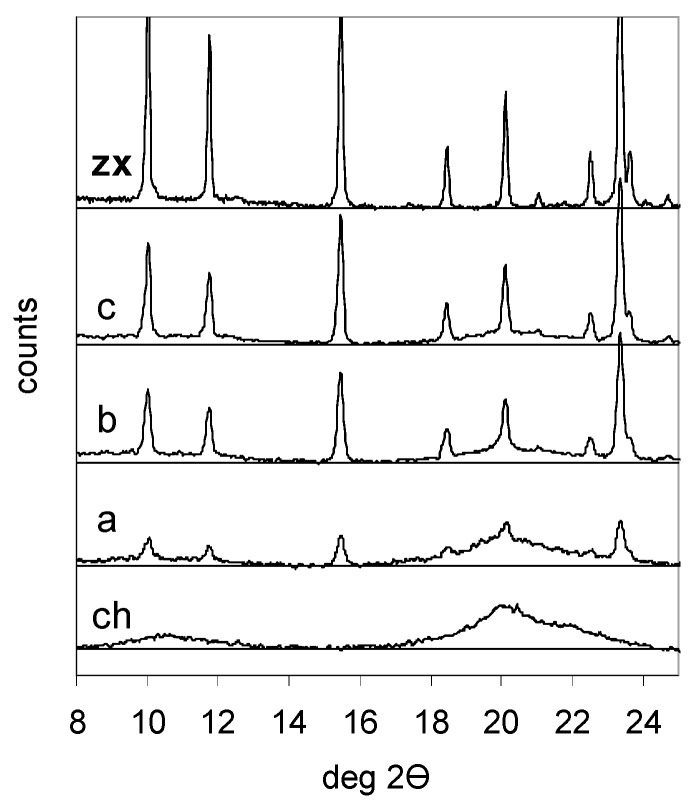
XRD patterns of chitosan (**ch**); chitosan-zeolite composites A (**a**); B (**b**) and C (**c**); and zeolite Na-X (**zx**).

In an attempt to preserve an aluminium-rich zeolite phase like zeolite Na-X in the composite, we choose much milder synthesis conditions. The zeolite was stirred for half an hour in chitosan solution with an acidic acid/glucosamine ratio 0.9. In Figure 1 the X-ray diffractograms of the composites of a chitosan aerogel prepared by the same procedure, and of the parent zeolite Na-X are reported. The XRD patterns of all composites show the characteristic peaks of zeolite X and the broad band of poorly crystallised chitosan around 20°2ϴ. When the amount of the zeolite increases (Table 1), the intensity of the diffraction peaks of chitosan decreases, as evidenced in Figure 1.

**Table 1:** Composition of the composites and properties of the zeolitic component.

Sample	Zeolite/Chitosan Mass Ratio		Cell Parameter (nm)	Zeolite X	Crystallite Size (nm)
	Synthesis	Composite		Si/Al	
Zeolite X			2.497	1.29	95
Composite A	0.3	0.27	2.497	1.30	25
Composite B	1.0	0.94	2.498	1.27	38
Composite C	1.5	1.45	2.500	1.24	35

The formation of composites induces extremely small changes in the cell size, which moves from 2.497 nm of the parent zeolite Na-X to 2.500 nm for composite C. The observed change of cell parameter corresponds to a slight increase of the aluminium content of the zeolite, whose Si/Al ratio moves from 1.29 to 1.24. This result clearly indicates that the mild conditions used in the encapsulation process have indeed prevented any dealumination. The slight desilicification observed has likely taken place in the alkaline solution of the gelling step.

The thermogravimetric (TG) data reported in Table 1 indicate that the zeolite/chitosan ratio in the final composites is slightly lower than the ratio between initial reagents. This loss of mass and the observed desilicification correspond to a non-congruent partial dissolution of the zeolite in the gelling alkaline solution [74,75,76]. Some broadening of the diffraction peaks of the zeolite indicates a decrease of the size of the domains of coherent diffraction and is related to the dissolution process. The absence of the typical broad hump of amorphous aluminosilicate centered at 30°2ϴ suggests that no amorphisation of the zeolite has taken place.

**Figure 2:**
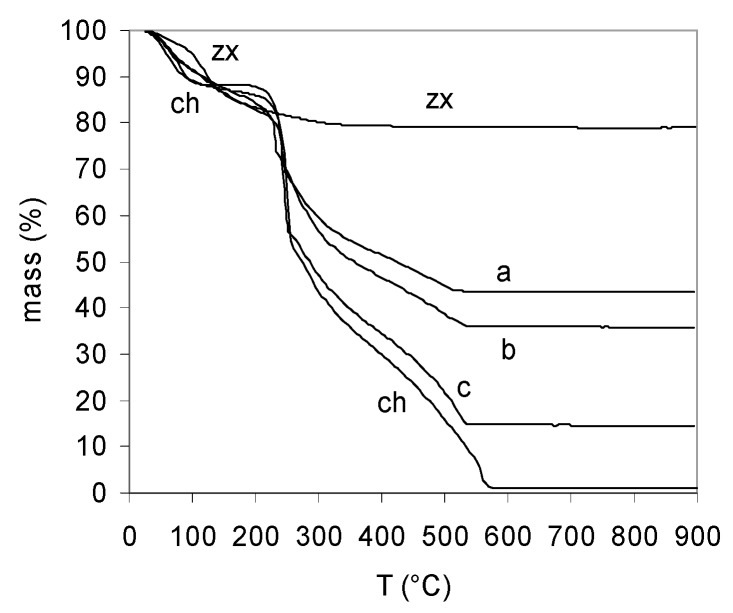
TG plots of aerogels of chitosan (**ch**), composites A (**a**); B (**b**); C (**c**) and zeolite X (**zx**).

Figure 2 illustrates the TG curves of chitosan and chitosan-zeolite composite aerogels (A, B, C) as well as of zeolite X. While zeolite X lose hydration water in a quite continuous way up to 400 °C, chitosan and composites presented a more complex pattern [77]. Water was lost at temperature lower than 140 °C Decomposition of the organic matrix suddenly started around 250 °C and continued at a more sedate pace until, at 600 °C, the organic part of the composites was completely removed and the residual mass corresponded to encapsulated inorganics.

**Figure 3:**
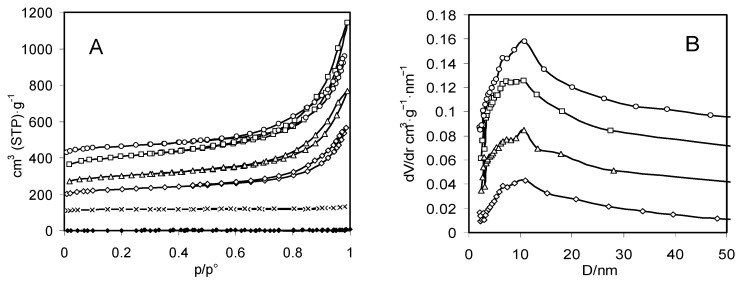
N_2_ adsorption-desorption isotherms (**A**) and pore size distributions (**B**) of zeolite X (crosses) and aerogels (void symbols) and xerogels (filled symbols) of chitosan (circles) and composites A (squares), B (triangles), and C (lozenges). Aerogel curves are shifted by 100 cm^2^ (STP)·g^−1^ in (**A**) and by 0.02 cm^3^·g^−1^·nm^−1^ in (**B**).

The N_2_ adsorption-desorption isotherms of zeolite X and of the aerogels and xerogels of chitosan and chitosan-zeolite composites are reported in Figure 3A and the mesopore size distributions of the aerogels are reported in Figure 3B. Values of surface area and pore volume calculated from the isotherms are reported in Table 2.

**Table 2:** Textural properties of isolated components and composites.

Sample	Zeolite Mass Fraction	Surface Area (m^2^·g^−1^)	Micropore Volume (cm^3^·g^−1^)	Mesopore Volume (cm^3^·g^−1^)
Chitosan aerogel	0	230	0	0.63
Composite A aerogel	0.21	390	0	0.89
Composite B aerogel	0.48	360	0.04	0.63
Composite C aerogel	0.59	480	0.11	0.44
Zeolite X	1	450	0.18	0.01
Chitosan xerogel	0	4	0	0.004
Composite B xerogel	0.48	1	0	0.002
Composite C xerogel	0.59	4	0	0.006

The isotherm of zeolite Na-X is type I in the IUPAC classification, as expected by a microporous adsorbent. The micropore volume of 0.18 cm^3^·g^−1^ is lower than the value expected for the porosity of faujasite, corresponding to the retention of some hydration water of the cations at the low outgassing temperature of 50 °C [78].

The isotherm of chitosan aerogel is type IV in the IUPAC classification and corresponds to a surface area of 230 m^2^·g^−1^ and a very broad pore size distribution with two maxima around 7 and 11 nm. The pore size distribution continues beyond 50 nm, indicating a mesoporous/macroporous texture of the chitosan aerogel. This mesopore size distribution remains unchanged when the zeolite is encapsulated in the composites. However, the presence of the zeolite modifies the pore volume and the surface area of the composites in a way which cannot be just attributed to the plain addition of the properties of isolated zeolite and chitosan. 

**Figure 4:**
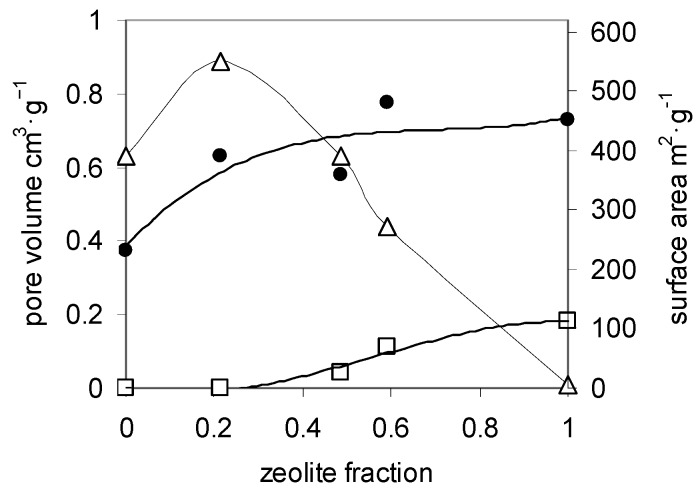
Texture of chitosan and composite aerogels *vs.* zeolite content. Mesopore (void triangles) and micropore (void squares) volumes (left side Y axis) and surface area (filled circles, right side Y axis). The lines are guides for the eye.

Pore volume and surface area values of the composite aerogels are reported in Figure 4 as a function of the zeolite content. The encapsulation of 21% zeolite in the composite A does not correspond to the development of any microporosity, suggesting that a polysaccharide coating has blocked the porosity of the zeolite, or at least has prevented its dehydration. As already stated, chitosan and composite A aerogels share the same pattern of pore size distribution. However, the values of mesopore volume and surface area of the composite are about one and a half times the values measured for the pure polysaccharide and exceed the values of the best chitosan aerogels [79]. This increase has to be related to the presence of the zeolite component in the composite and can be tentatively attributed to an improvement of the rigidity of the fibrils of polysaccharide in the drying process.

When the amount of zeolite is further increased, as in composites B and C, some microporosity can be measured as far as a fraction of zeolite is no more occluded by chitosan. In the meantime, the mesopore volume linearly decreases following the decrease of the fraction of chitosan, whereas the surface area irregularly increases by the mixed effect of the decrease of the amount of chitosan and the increase of accessible zeolite in the composite. In the case of the xerogels of chitosan, it is well known that the shrinkage in the evaporative drying leads to a material without any porosity [25,80]. It is significant that also the xerogels of the three composites are non-porous, despite of the amount of zeolite present. This indicates that in all cases the microporosity of the zeolite has been completely blocked by evaporatively dried chitosan gel. 

**Figure 5:**
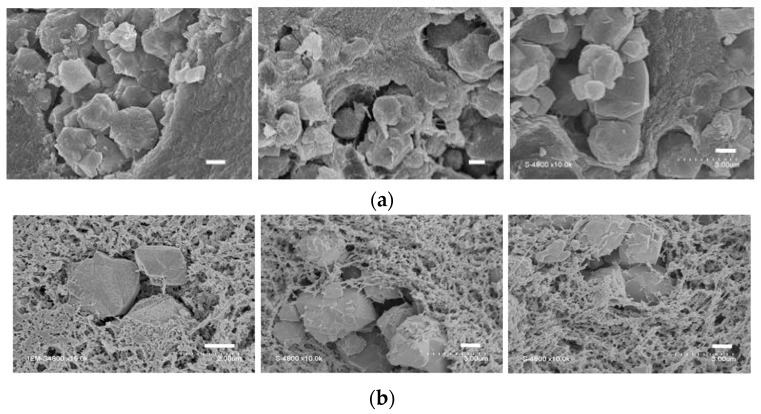
Scanning electron micrographs of xerogels (**a**) and aerogels (**b**) of composites A, B, and C from left to right. Scale bars 1 μm.

SEM micrographs of chitosan and chitosan-NaX composite xerogels and aerogels are shown in Figure 5. In all cases, zeolite X is present as loose aggregates of micrometric octahedral crystals. In the case of the xerogels, the zeolite crystals are included in cavities of a continuous polysaccharide matrix. It appears that, in the case of evaporative drying, the shrinkage of the polysaccharide gel has led to a physical separation between polymer and embedded zeolites. In the case of the aerogels, an open tridimensional network of polysaccharide fibrils can be observed and a good contact is retained between zeolite crystals and the chitosan maze. 

### 2.2. Adsorption of Copper

#### 2.2.1. Copper Sorption on Chitosan Gels

**Figure 6:**
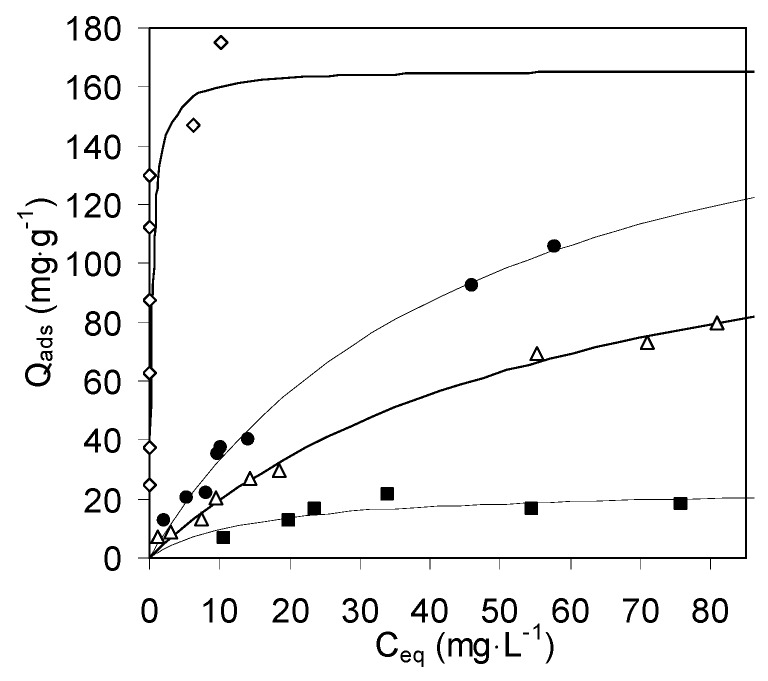
Cu(II) sorption on zeolite X (void lozenges), chitosan hydrogel (filled circles), aerogel (void trangles) and xerogel (filled squares). The lines are best-fit Langmuir curves.

The use of chitosan hydrogels implies severe problems of biodegradability of the wet material. As a consequence, gel beads have to be stocked in a dry form before their rehydration just before the use in the sorption device. In this context, the drying method and the corresponding effectiveness of restoration of the gel texture upon rehydration can be critical parameters for the sorption of metal cations. In this work, sorption of Cu(II) from aqueous solutions was carried out onto beads of chitosan hydrogel and in-situ rehydrated beads of supercritically dried aerogel and evaporatively dried xerogel. The sorption isotherms are reported in Figure 6, together with the sorption isotherm of Cu(II) on zeolite Na-X. The constants of the corresponding Langmuir best-fits are given in Table 3.

The affinity of chitosan for copper sorption is strongly influenced by the environment of the amino groups. The pKa of chitosan is around 6.3, so at pH 6 the glucosamino groups are largely protonated. However, copper cations can easily form complexes with protonated glucosamino groups by the reaction Cu^2+^ + 2R-NH_3_^+^ + 2H_2_O = [Cu(R-NH_2_)_2_]^2+^ + 2H_3_O^+^, especially favoured between pH 5.8 and 6.3 [81]. This kind of complexation implies the insertion of Cu(II) between two chitosan chains [82].

**Table 3:** Langmuir constants for the adsorption of Cu(II) ions ^a^.

Sample	Zeolite Mass Fraction	Hydrogel		Aerogel		Xerogel	
		Q_max_ (mg·g^−1^)	K (L·mg^−1^)	Q_max_ (mg·g^−1^)	K (L·mg^−1^)	Q_max_ (mg·g^−1^)	K (L·mg^−1^)
Chitosan	1	190% ± 10%	0.021% ± 18%	139% ± 9%	0.016% ± 16%	24% ± 23%	0.07% ± 72%
Composite A	0.21	155% ± 10%	0.083% ± 30%	168% ± 18%	0.025% ± 33%	9.4% ± 14%	0.29% ± 63%
Composite B	0.48	111% ± 16%	0.15% ± 62%	113% ± 14%	0.040% ± 34%	12.7% ± 9%	0.38% ± 72%
Composite C	0.59	83% ± 9%	0.28% ± 50%	110% ± 13%	0.065% ± 38%	15.6% ± 10%	3.5% ± 100%

^a^ To be compared with values for zeolite Na-X: Q_max_ 166 mg**·**g^−1^, K 2.6 ± 2.6 L**·**mg^−1^.

The amount of glucosamino groups in pure dry chitosan is 6.2 mmol·g^−1^. Our experimental data on chitosan hydrogel indicate, at saturation of the theoretical Langmuir monolayer, a maximum copper sorption of 190 mg·g^−1^, corresponding to 2.97 mmol·g^−1^. This suggests that a mechanism of complexation of one Cu(II) cation by two amino groups is fully operative and that copper cations can freely penetrate inside the hydrogel fibrils.

However, the drying method significantly affects the copper sorption capacity. Indeed, the results showed a significant decrease of nearly 25% in the case of aerogel and nearly 90% in the case of xerogel chitosan beads. It has been observed that chitosan gels undergo a partial shrinkage upon supercritical drying [79]. It is a reasonable hypothesis that a decrease of accessibility due to shrinkage can account for the lower Cu(II) capacity of the rehydrated chitosan aerogel.

In the case of the chitosan xerogel, the striking decrease of the Cu(II) capacity seems to be accompanied by a strong increase of the Langmuir K constant, which would correspond to an increased affinity of the adsorbent for Cu(II). This effect probably corresponds to an artefact related to the very limited accessibility of the xerogel. Very likely, the immersion of the beads in the solution brings about the swelling of a thin outer layer, which is the only part of the xerogel which becomes accessible to the solution and is saturated by Cu(II) at quite low concentration. Such an effect can easily explain the adsorption capacities reported in the literature for dried chitosan, not higher than our data [5,29]. 

#### 2.2.2. Sorption of Cu^2+^ on Zeolite Na-X

The effectiveness of faujasite-type zeolites in the removal of copper from aqueous solutions by exchange of the sodium cations is recognized [83,84]. The experimental equilibrium isotherm for adsorption of copper on Na-X reported in Figure 5 shows a steep initial slope and reaches a plateau at a low residual metal concentration. The shape of the isotherm corresponds to a high affinity for Cu(II) and a corresponding high K of the Langmuir equation. In Figure 5, the Langmuir isotherm has been drawn to saturation at the experimental exchange capacity of Na-X (166 mg**·**g^−1^) [85] (Table 3). 

Compared to chitosan hydrogel, zeolite X has a slightly lower capacity and a much higher affinity for copper sorption. Despite this higher affinity, the use of zeolite X alone in wastewater treatment is limited by the need of retaining the zeolite crystals in a matrix. However, the interest of a high affinity for the sorption of trace amounts of metal cations suggests a potential interest of zeolite X for the improvement of the properties of chitosan-based sorbents.

#### 2.2.3. Sorption Isotherms of Cu^2+^ on Composites Chitosan-Zeolite X

**Figure 7:**
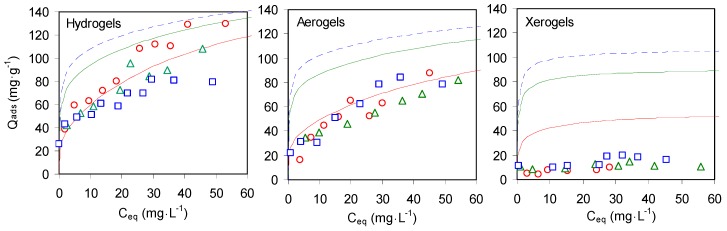
Copper sorption isotherms on hydrogels, aerogels and xerogels of composites A (21% zeolite, red void circles), B (48% zeolite, green void triangles) and C (59% zeolite, blue void squares) and pure chitosan (black filled circles and Langmuir best-fit line).

Copper sorption experiments were performed with the same methodology on composites chitosan-zeolite X. The three composites A, B and C, prepared with different mass ratios zeolite/chitosan (0.3, 1 and 1.5, respectively) were used in their three states: hydrogel, aerogel and xerogel. Figure 7 shows the experimental equilibrium isotherms of copper sorption. The constants of the corresponding Langmuir best-fits are given in Table 3.

In the case of the hydrogels, all composites presented a copper sorption at low concentration much higher than the sorption on pure chitosan (full line in Figure 7, left hand graph). At higher concentration, the sorption on the zeolite-poor composite A (void circles) was virtually parallel to the sorption curve on pure chitosan. At higher zeolite content (void triangles and squares), the sorption isotherms of the composites became flatter and did not exceed the sorption capacity of pure chitosan at high copper concentration. The parameters of the Langmuir plots reported in Table 3 parallel these observations. An increase of the Langmuir affinity constant with the amount of zeolite present justifies the better effectiveness of composite sorbents at low concentrations. However, the Langmuir Q_max_ steadily decreases with the amount of zeolite in the composite and in the zeolite-rich composite C it is less than the half of the Q_max_ expected from a mechanical mix with the same chitosan/zeolite ratio. The results suggest that, despite a fraction of zeolite provides the observed high affinity at low concentration, the addition of further amounts of zeolite does not improve the sorption properties.

In the case of the aerogels, all composites present a similar sorption behaviour (Figure 7, central graph). The sorption at low concentration is higher than the sorption on the aerogel of pure chitosan (full circles and line) and at higher concentration the sorption trend of the composites parallels the chitosan isotherm at slightly higher values. Indeed, an increase of the Langmuir affinity constant with the zeolite fraction (Table 3) is partially compensated by a decrease of the Q_max_. 

Figure 7 allows visually comparing the sorption isotherms of aerogels and hydrogels. The sorbed amounts on aerogels are patently lower that the amount sorbed on the corresponding hydrogels, especially at the lowest equilibrium concentrations. The Langmuir Q_max_ of Table 3 could seem in disagreement with this visual observation, as they are quite similar for corresponding aerogels and hydrogels. This apparent contradiction is solved when the Langmuir affinity constants of the same table are taken into account, as they are systematically lower for the aerogels than for the hydrogels. This indicates that a potential high sorption capacity is only reached at not realistical high concentrations. Globally, the sorption trends correspond to the need for a higher concentration in solution to make accessible the sorption sites in the aerogels than in the hydrogels of the composites.

As already observed in the case of the textural properties, the method of drying is critical to the preservation of the sorption capacity. The sorption isotherms of the composite xerogels strictly adhere to the same pattern as the isotherm of the xerogel of pure chitosan, with a limited sorption at low concentration and extremely limited further sorption beyond a concentration of 10 g cm^−3^. These Langmuir Q_max_ are an order of magnitude lower than in the case of the aerogels or the hydrogels, whereas the Langmuir affinity constant increases up to zeolite-like values. This effect, already observed for the xerogel of pure chitosan, corresponds to the fast saturation at low concentration of just a limited swellable outer rim of the sorbent. This result is in excellent agreement with literature reports of the sorption behaviour of dried chitosan-zeolite composites [62,64].

## 3. Experimental Section

### 3.1. Materials

Chitosan (medium molecular weight, viscosity 32cps) and zeolite Na-X were used as purchased from Sigma-Aldrich (St. Louis, MO, USA). Copper nitrate Cu(NO_3_)_2_·3H_2_O was supplied by ACROS (Fisher Scientific, Illkirch-Graffenstaden, France). 

The degree of acetylation (DA) of chitosan was evaluated by the Miya infrared spectroscopy method [86] using a Vector 22 FT-IR spectrometer (Bruker, Billerica, MA, USA). DA was also determined using the Hirai H^1^-NMR method [87]. Spectra were recorded on a Bruker ASX 400 (400 MHz) spectrometer. Both measurements indicated a degree of acetylation of 10%.

The accessibility of the chitosan primary amine functions was investigated by the formation of 2-methylformylbenzimidaldimine-Schiff base upon treatment with 2-methylformylbenzimidaldehyde. Quantitative GC analysis of the remaining 2-methylformylbenzimidaldehyde in the solution gives the fraction of reacted amino groups, which corresponds to the accessible sites. 

### 3.2. Preparation of Chitosan Beads

Chitosan (4.5% *w*/*w*) was totally dissolved by stirring at room temperature in 2% acetic acid solution. This solution was dropped into a NaOH solution (4 N) through a 0.8 mm syringe needle. The chitosan hydrogel beads were stored in the alkaline solution for 2 h and then filtered and washed with water. Two different routes were followed to dry the microspheres. One consisted of dehydrating the chitosan gel beads by evaporation at room temperature. This procedure leads to a xerogel. The second consists of drying the chitosan beads under supercritical CO_2_ conditions [25,80]. This technique leads to aerogel beads. 

### 3.3. Preparation of Chitosan-Zeolite Composites

Chitosan-zeolite composites have been prepared by the encapsulation method. The procedure was based on dispersing zeolite X crystals in chitosan-acetic acid solution obtained by dissolving chitosan 3% *w*/*w* in 1% acetic acid solution. Different zeolite/chitosan mass ratios were used: 0.3, 1 and 1.5. The corresponding composites are named A, B, and C, respectively. After homogenisation, the suspensions were added drop by drop to a NaOH solution (4 N). Beads of hydrogel were obtained and dried to xerogels and aerogels as described previously for chitosan beads.

### 3.4. Characterization of Materials

Nitrogen adsorption was performed at 77 K in an ASAP 2010 volumetric instrument (Micromeritics, Norcross, GA, USA). The samples were outgassed at 50 °C prior to the adsorption measurement until a 3 × 10^−5^ Torr static vacuum was reached. The outgassing temperature has been chosen in order to preserve the texture of the more temperature-sensitive polysaccharide aerogels. The surface area was calculated by the Brunauer-Emmett-Teller (BET) method [88]. Micropore volume was evaluated by the α-S method using a standard isotherm measured on non-porous silica [89]. The mesopore volume was evaluated as the adsorbed volume at p/p° 0.95 minus the micropore volume. The mesopore size distribution was evaluated by a DFT kernel proposed by Neimark [90]. 

Powder X-ray diffraction (XRD) patterns were collected on a Bruker AXS D-8 diffractometer with Cu Ka radiation on wafers obtained by compression at 0.7 GPa. The framework aluminium content of the zeolite has been evaluated from the unit cell parameter by the Kerr’s correlation [71,72,73]. Crystallite size has been evaluated by the Williamson-Hall method. 

The thermal stability and the organic contents of the dried solids were determined by thermogravimetric analysis. The samples were heated in air flow at 10 °C**·**min^−1^ up to 900 °C in a TG 209 C thermal balance (Netzsch, Selb, Germany). The amount of zeolite present in the composites was evaluated from the residual mass at 900 °C by taking allowance of the expected hydration water of the zeolite (26.5% of the dry mass).

SEM micrographs were recorded on a S4500 microscope (Hitachi, Chiyoda, Japan).

### 3.5. Copper Sorption Studies

A known amount of chitosan or composite chitosan-NaX (10 mg) was added to a given volume of copper nitrate solution (50 mL) at known concentrations (10–80 ppm) and pH = 6. This pH, often indicated as the preferred one for Cu(II) sorption on chitosan [5,91], was selected to optimise the stability of chitosan and zeolite in the sorption solution [92,93]. The sorption batches were continuously shaked on a mechanical shaker (200 rpm) at 30 °C. Equilibrium was reached in 6 days for copper adsorption on chitosan and in 3 days for the chitosan-NaX composites. Samples were finally collected and filtered. Copper content in the filtrate was measured by atomic absorption spectrometry in an AA 220 apparatus (Varian, Palo Alto, CA, USA).

The equilibrium uptake capacity of the adsorbents at each concentration was calculated according to Equation (1):

(1) $$q_{\text{ads}}=\frac{\left(C_{0}-{\text{ C}}_{\text{eq}}\right)\times \text{ V}}{m}$$

where q_ads_ is the amount adsorbed per unit mass of adsorbent (mg**·**g^−1^), C_0_ and C_eq_ are, respectively, initial and equilibrium concentrations of metal ion (mg**·**L^−1^), m is the mass of adsorbent (g) and V is the volume of solution in litters. The sorption data were parametered using the Langmuir Equation (2):

(2) $$q_{\text{ads}}=\frac{C_{\text{eq}}Q_{\text{max}}}{\frac{1}{K}+{\text{ C}}_{\text{eq}}}$$

where Q_max_ is the sorption capacity (mg**·**g^−1^) of Cu(II) at saturation of the Langmuir theoretical monolayer and K is the Langmuir adsorption constant (L**·**mg^−1^) corresponding to 1/C_half capacity_. Best-fit of the Langmuir equation and statistical reliability of the parameters were calculated by Prism 6 software (GraphPad, San Diego, CA, USA).

## 4. Conclusions

The exchange capacity of zeolite X as sorption-boosting charge in chitosan-zeolite composites has been preserved by controlling the encapsulation conditions. Successful dissolution of chitosan in solutions with acetic acid/chitosan ratios lower than one has allowed us to avoid any dealumination of the zeolite. Subsequent gelation of the zeolite suspensions in alkaline solution has allowed the preparation of composite beads with different zeolite/chitosan ratios.

The need to store the composites in a dry state before their use as sorbents for the treatment of contaminated aqueous solutions has prompted us to study the most suitable drying methods. Evaporative drying led to non-porous xerogels unable to recover any significant accessibility when rehydrated in the sorption solutions. On the contrary, supercritical CO_2_ drying has allowed the formation of highly porous aerogels with significant Cu(II) sorption capacity when rehydrated. Indeed, when aerogels of chitosan are used for Cu(II) sorption from aqueous solution at pH 6, the Langmuir Q_max_ capacity of the hydrogels is essentially retained, with a small decrease of the Langmuir affinity constant.

Despite the coating of chitosan that often prevents easy access to the microporosity of the zeolite, the present zeolite encapsulation process leads to a stabilisation of the mesoporous network of chitosan, with a significant increase of surface area, mesoporous volume and sorption capacity when just a limited amount of zeolitic charge is incorporated. Chitosan aerogels with a limited amount of encapsulated zeolite X represent a storable sorbent for Cu(II) cations which retain 90% of the 190 mg**·**g^−1^ capacity of freshly prepared chitosan hydrogels. Moreover, the presence of a limited amount of zeolite significantly increases the affinity of the sorbent for Cu(II) at low concentration in solution, an especially desirable properties for secondary treatments of polluted streams.
